# A175 IMPACT OF THE COVID-19 PANDEMIC ON CANADIANS WITH CELIAC DISEASE

**DOI:** 10.1093/jcag/gwad061.175

**Published:** 2024-02-14

**Authors:** J A King, C McAulay, M Secord, M Pinto-Sanchez, J Turner, D Gidrewicz, S Case, D Duerksen

**Affiliations:** University of Calgary, Calgary, AB, Canada; Canadian Celiac Association, Mississauga, ON, Canada; Canadian Celiac Association, Mississauga, ON, Canada; McMaster University, Hamilton, ON, Canada; University of Alberta, Edmonton, AB, Canada; University of Calgary, Calgary, AB, Canada; Canadian Celiac Association, Mississauga, ON, Canada; University of Manitoba, Winnipeg, MB, Canada

## Abstract

**Background:**

The COVID-19 pandemic affected various healthcare services, including primary care and management of certain health conditions. There is limited data on how the pandemic impacted those with celiac disease (CD).

**Aims:**

Explore the implications of the COVID-19 pandemic on CD diagnosis and management.

**Methods:**

The State of Celiac Disease in Canada Survey was made available online by Celiac Canada in December 2022. Over 75 questions were included, covering diagnostic pathways, COVID-19 factors such as infection and vaccination, perceptions around COVID-19, and gluten-free food access during the pandemic. Analysis was restricted to those who responded to having CD, dermatitis herpetiformis, or gluten ataxia.

**Results:**

A total of 6052 participants were included. A total of 2320 (38.3%) reported having tested positive for COVID-19 at some time during the pandemic. Among those with an infection at some point, 93.8% tested positive after already being diagnosed with CD. Just under 2% of those testing positive for COVID-19 required hospitalization, of which 10% were admitted to the intensive care unit. Vaccination against COVID-19 was reported by 4695 (77.6%) individuals, although 1157 (19.1%) did not provide a response to this specific question. This did not differ significantly by gender (p = 0.901), although it did across age groups (p ampersand:003C 0.001), with a mean age of 54.2 years who were vaccinated compared to a mean age of 45.7 years who were not vaccinated. 718 (11.9%) of individuals felt they were at higher risk of COVID-19 complications due to having CD; however, 1290 did not respond. Reported delays from symptoms to diagnosis did not increase among those diagnosed during the pandemic compared to those diagnosed before. Most respondents did not feel the pandemic made it more challenging to follow the gluten-free diet, and just over half (52.8%) did not feel it was harder to obtain or access gluten-free food during this time. However, almost three-quarters (73.4%) noted an increase in the cost of gluten-free food during the pandemic – this meant adjusting finances in other areas to afford gluten-free food (28.5%), changing the type of food purchased (19.3%), or accessing a food bank (0.7%).

**Conclusions:**

A significant number of patients with CD reported COVID-19 infections, but less than 2% had serious outcomes. This survey identified gaps in our understanding of COVID-19 vaccination in patients with CD and highlighted some adverse impacts of the pandemic on the costs of gluten-free foods.

**Funding Agencies:**

Celiac Canada (FKA Canadian Celiac Association)

